# Draft Genome Sequence of a *Leptospira interrogans* Strain Isolated from the Urine of an Asymptomatic Dog in Thailand

**DOI:** 10.1128/genomeA.01140-17

**Published:** 2018-01-25

**Authors:** Alongkorn Kurilung, Chantisa Keeratipusana, Prapat Suriyaphol, Nuvee Prapasarakul

**Affiliations:** a Department of Veterinary Microbiology Faculty of Veterinary Science, Chulalongkorn University, Bangkok, Thailand; b Bioinformatics and Data Management for Research Unit, Office for Research and Development, Faculty of Medicine Siriraj Hospital, Mahidol University, Bangkok, Thailand

## Abstract

In 2014, *Leptospira interrogans* strain CUDO8 was isolated from the urine of an asymptomatic dog in Thailand. Here we report the draft genome sequence of this pathogenic bacterium.

## GENOME ANNOUNCEMENT

Leptospirosis is an important zoonotic disease caused by infection with pathogenic spirochetal bacteria in the genus *Leptospira* (1). The disease is especially common in tropical regions, including Thailand. Most mammalians are infected with *Leptospira* spp. and show a wide range of clinical presentations varying from acute to chronic infections (2). Animals with chronic infections act as carriers, as they harbor leptospires in the convoluted tubules of the kidneys and shed them into the environment via their urine (3). To date, there is limited information about the genomes of *Leptospira* isolated from asymptomatic animals, and few studies have investigated host adaptation in chronic infections. Consequently, analysis of the genome sequence of *Leptospira* isolated from an asymptomatic dog might provide important clues about mechanisms of host adaption in these bacteria.

*Leptospira interrogans* strain CUDO8 was collected from the urine of an asymptomatic dog in Nan province, Thailand, in 2014 and was identified by phylogenetic analysis of the 16S rRNA gene (*rrs*) (4). Purified leptospires were cultured in liquid EMJH medium, and their DNA was extracted and sequenced using the MiSeq platform with 251 paired-end run cycles (Illumina, Inc., USA). *De novo* assembly was carried out using the A5-MiSeq pipeline (5) and comprised read trimming, base error correction, contig assembly, and scaffolding. Scaffolds were further ordered and oriented by ABACAS (6) using the *L. interrogans* serovar Lai strain 56601 as a reference, and the gaps were closed using IMAGE (7). The draft genome sequence was annotated by using rapid prokaryotic genome annotation (PROKKA) (8) and Rapid Annotations using Subsystems Technology (RAST) version 4.0 (9).

A total of 83 scaffolds were obtained after the assembly process, with 100× coverage. The length of genome was ~4.9 Mbp with an *N*_50_ value of 165,528, and a G+C content of 35%. With the use of PROKKA annotation, the strain CUDO8 was predicted to have 4,013 putative protein-coding sequences (CDSs) with 38 tRNAs and 3 rRNAs (5S [*n* = 1], 16S [*n* = 1], and 23S [*n* = 1]). Moreover, RAST identified 312 subsystems involved in RNA metabolism, cofactors and vitamins, amino acids and derivatives, cell wall and capsule components, and motility and chemotaxis.

### Accession number(s).

The draft genome sequence of *L. interrogans* strain CUDO8 has been deposited at DDBJ/ENA/GenBank under the accession number NKYG00000000; the 83 scaffolds have been deposited under the GenBank accession numbers NKYG01000001 to NKYG01000083. The version described in this paper is NKYG01000000.
